# Pseudo-Anterior Interosseus Nerve Syndrome: A Case Report and a Review of Clinical Signs, Pathology and Functional Anatomy of the Precision Grip

**DOI:** 10.7759/cureus.15180

**Published:** 2021-05-22

**Authors:** Hassan Kesserwani

**Affiliations:** 1 Neurology, Flowers Medical Group, Dothan, USA

**Keywords:** median nerve injury, unilateral weakness, power loss, grip weakness, neuropathy

## Abstract

Precision grip, a prehensile function of humans, is exacted through the action of the median nerve and its main tributary, the anterior interosseus nerve (AIN). In the forearm, the AIN can be subject to nerve entrapment by tendinous and fibrous arches or accessory and variant muscles. It is also vulnerable to trauma of the upper arm and forearm. To the neurologist, an isolated neuritis or an immune-mediated medial cord or lower trunk brachial plexopathy (Parsonage-Turner syndrome) is the usual mode of presentation. When the spread of muscle weakness is beyond the territory of the AIN, the syndrome is referred to as a pseudo-AIN. The AIN is grouped into fascicles that are compartmentalized separately from the median nerve proper, and trauma in the upper arm may selectively involve the AIN. We present a case of pseudo-AIN following elbow arthroscopic surgery and outline the pathology, clinical signs, and functional anatomy of the AIN and the precision grip.

## Introduction

The median nerve of the forearm supplies the muscles that arise from the medial epicondyle, the flexor digitorum superficialis (FDS) or sublimis and the flexor-pronator group of muscles, namely the pronator teres (PT), the flexor carpi radialis (FCR), and the palmaris longus (PL). After the median nerve emerges from between the two heads of the pronator teres, the anterior interosseus nerve (AIN) arises from the median nerve about a palm-breadth below the lateral epicondyle, coursing underneath the fibrous arch of the FDS, after which it penetrates the belly of the FDP. The AIN is a pure motor nerve, travelling on the surface of the interosseous membrane, and it supplies three muscles; the flexor pollicis longus (FPL), the radial portion of the flexor digitorum profundus (FDP), and the pronator quadratus (PQ). Proximal to the radial styloid the palmar cutaneous branch, which supplies the skin overlying the thenar eminence, is the last branch of the median nerve in the forearm. The median nerve, FPL, FDP, and FDS tendons travel through the fibro-osseus carpal tunnel [1].

At the outset, we should note that the FDS flexes the metacarpophalangeal (MCP) joints and proximal interphalangeal joints (PIP) of all the fingers except the thumb, which is flexed by the FPL. Flexion of the distal interphalangeal joints (DIP) of the index and middle finger is carried out by the radial portion of the FDP which is supplied by the AIN. The ulnar portion of the FDP, which flexes the DIP joints of the ring and little fingers, is supplied by the ulnar nerve [2]. Variations of nerve supply do occur but studies are limited. In a study of 20 cadavers by Bhadra et al., the radial portion of the FDP to the index and ring fingers had exclusive innervation by the AIN and the ulnar nerve supplied the ulnar portion of the FDP to the middle, ring, and little finger in 75% cases, meaning that the middle finger has dual innervation in 75% of cases. In 20% of cases, the nerve supply of the AIN is to the radial portion of the FDP (index and middle fingers) and an ulnar portion of the FDP (ring and little fingers), an even split. In the remaining 5% of cases, the index finger was exclusively innervated by the AIN, and the rest of the fingers (middle, ring, and little fingers) are supplied by the ulnar nerve [3].

The AIN can be assessed by the pinch maneuvre. During a normal pinch, the attitude of the index finger and thumb form the letter "O". Normally the pulp of the index finger meets the pulp of the thumb, with perfect opposition. With an AIN paralysis, there is a failure of flexion of the thumb at the interphalangeal joint and the index finger at the distal interphalangeal joint. There is compensatory hyperflexion of the more proximal joints with the displacement of the pulp of the index finger posteriorly on the thumb. Useful clinical pearls include: (1) the PQ function can be assessed through eliminating the pronator teres pronator action by fully flexing the elbow by eliminating the function of the humeral head of the pronator trees; and (2) with an AIN injury, the pronator teres, supplied by the median nerve, shows no evidence of denervation by electromyography [4].

The disease of the AIN can be due to inflammation, nerve entrapment, or trauma. The AIN can be involved in isolation, as in a mononeuritis, or part of a broader brachial plexitis with the involvement of muscles outside the distribution of the AIN. The brachial plexitis typically involves the medial cord of the brachial plexus with involvement of the medial cutaneous nerve of the forearm, possibly spread to the upper trunk of the brachial plexus with shoulder girdle weakness [5,6]. Nevertheless, pure involvement of the AIN should be restricted to the triad of thumb flexion weakness, weakness of DIP flexion of the index and/or middle fingers, and weakness of the PQ as demonstrated by forearm pronation weakness with full elbow flexion. The spread of muscle weakness outside the AIN territory is often referred to as pseudo-AIN syndrome. Multiple observers have noted that the fibres destined to become the AIN separate as a distinct bundle of fascicles prior to take-off from the median nerve. This compartmentalization can lead to a pseudo-AIN syndrome from an injury, inflammation, or entrapment of the median nerve proper with median sensory involvement [7-9].

There are instances where the ulnar-innervated small muscles of the hands (adductor pollicis, abductor digiti minimi, the first, second, and third interossei) manifesting as weakness of the finger spreaders and adduction of the thumb, are involved with an AIN lesion. This can happen with the Martin-Gruber anastomosis, whereby these muscles are supplied by a branch of the median nerve and not the ulnar nerve. This is known as a median-to-ulnar anastomosis and is seen in 15% of forearms. This can be recognized electrophysiologically by a greater proximal-than-distal compound muscle action potential (CMAP) recording of the abductor pollicis brevis (APB) when stimulating the median nerve at the ante-cubital fossa when compared to the wrist. This is due to the dual activation of both median and ulnar motor nerve fascicles at a site proximal to the anastomosis [10].

## Case presentation

A 62-year-old woman with a long-standing history of rheumatoid arthritis woke up the day after her left elbow arthroscopic surgery (debridement) with weakness of the non-dominant left hand. She noted weakness of grip and pinch with numbness (novocaine-like) of the left index finger and thumb. By the sixth month, she was still unable to zip-lock, grasp a necklace, manipulate an earring or hold utensils in the kitchen. No cervical radicular symptoms or other constitutional symptoms such as fever, fatigue, or myalgia were noted.

Past medical history is significant for a 20-year history of rheumatoid arthritis treated with methotrexate 7.5 milligrams (mg) per week, hydroxycholoroquine 300 mg daily, and once-monthy subcutaneous sarilumab, an "interleukin-6 antagonist". For arthritic pain, she takes celecoxib 200 mg twice daily and gabapentin 300 mg twice daily.

The pertinent neurologic examination reveals normal power in all the extremities except the left arm. The grading of muscle power of the left arm is listed below using the medical research council grading scale (MRC), (Table 1).

**Table 1:** Grading of power of the muscles of the left arm, using the medical research council (MRC) scale.

	Power grade
Left deltoid	5
Left biceps brachii	5
Left triceps	5
Left extensor carpi radialis longus	5
Left extensor digitorum communis	5
Left pronator teres	5
Left flexor carpi radialis	5
Left flexor digitorum superficialis	3
Left flexor pollicis longus	1
Left pronator quadratus	4
Left abductor pollicis brevis	2
Left interossei	5
Left adductor pollicis	5
Left flexor digitorum profundus to index finger	0
Left flexor digitorum profundus to ring finger	2

**Figure 1:**
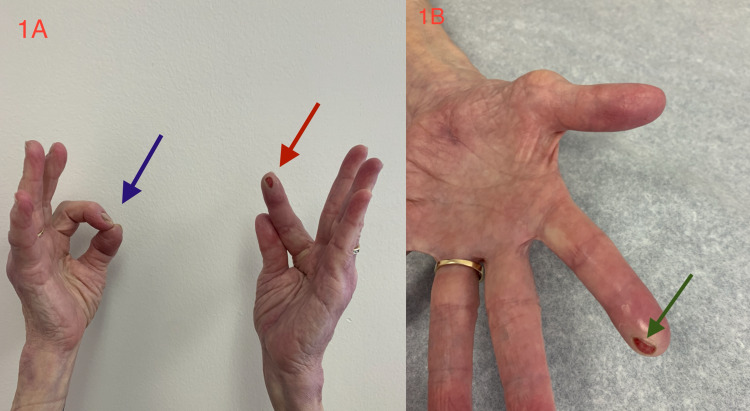
1 A- Right hand- normal formation of "O" symbol (blue arrow). Left hand - abnormal formation of pinch "O" sign due to lack of flexion of thumb (flexor pollicis longus) and index finger (radial half of flexor digitorum profundus), (red arrow). 1 B- ulceration of tip of index finger due to loss of sensation (green arrow).

The weakness of the left FPL, the distal PIP joint of the index finger (radial half of FDP), MCP joint, and proximal PIP (FDS) is demonstrated below by the inability to form an "O" gesture. The pattern of weakness reflects the weakness of the AIN-innervated muscles (radial half of FDP), FPL, and PQ, with extension beyond the AIN territory, with involvement of median nerve-innervated muscles (FDS and APB). Atrophy of the APB was noted with subtle wasting of the forearm muscles (FDS and FDP). Deep tendon reflexes were diminished throughout but were enhanced by the Jendrassik maneuvre, probably reflecting the atrophy of the muscles around her arthritic joints and loss of muscle spindles. There was a complete absence of pin-prick and temperature of the left thumb and index finger as demonstrated below by the anesthetic ulceration of the left index finger. The AIN, being a motor nerve, implies that the sensory loss is due to median nerve involvement (Figure 1).

**Figure 2:**
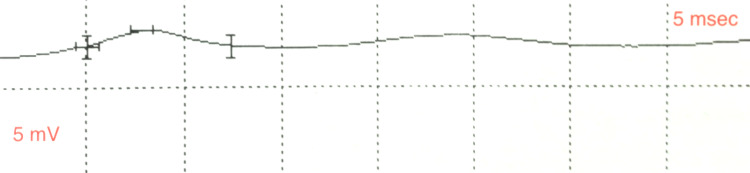
Nerve conduction study of the left flexor pollicis longus muscle shows a low amplitude of 0.833 mV, normal is 4 mV or above. Millivolt (mV), milliseconds (msec), time on the abscissa.

A nerve conduction study revealed the absence of the left APB compound muscle action potential (CMAP) with the absence of the median sensory nerve action potential. The FPL CMAP was reduced to 0.833 milliVolts (mV), lower limit of normal being 4 mV, implying AIN involvement (Figure 2).

The left ulnar motor amplitude (recording from the first dorsal interosseus) and the left ulnar sensory amplitude, latencies, and velocities were normal. The left radial motor amplitude (recording from the extensor indicis proprius) and the left radial sensory amplitude, latencies, and velocities were normal. The left medial antebrachial cutaneous sensory amplitude was also normal.

Acute-on-chronic denervation of median-nerve innervated and AIN-innervated muscles are displayed below (Table 2).

**Table 2:** Electromyography of left arm muscles demonstrating acute-on-chronic denervation of median and anterior interosseus-innervated muscle. Note the absence of nascent motor units, implying a lack of re-innervation. Fibrillations and positive waves are graded as follows: 1+ = transient with needle movement, 2+ = occasional, at rest in more than two sites, 3+ = present, at rest in most sites, 4+ = abundant, almost filling screen at all sites.

	Spontaneous activity	Motor unit morphology	Recruitment	Interference pattern
Left pronator teres	None	Normal	Full	Full
Left flexor carpi radialis	None	Normal	Full	Full
Left flexor digitorum superficialis	2+	Large polyphasic long duration motor units	Reduced	Reduced
Left palmaris longus	None	Normal	Normal	Normal
Left pronator quadratus	Unable to locate			
Left flexor pollicis longus	2+	Large polyphasic long duration motor units	Reduced	Reduced
Left radial half of flexor digitorum profundus	2+	Large polyphasic long duration motor units	Reduced	Reduced
Left abductor pollicis brevis	2+	Large polyphasic long duration motor units	Reduced	Reduced

The constellation of motor and sensory findings outlined above implies a predominantly AIN lesion with some spread to median-innervated muscles and sensory involvement which parallels the definition of a pseudo-AIN. The most proximate cause being nerve injury arising from arthroscopic manipulation of the elbow joint. The patient attended physical and occupational therapy but by the sixth month, the motor and sensory deficits remained static. Options to consider include tendon transfer from the brachioradialis tendon to the FPL tendon in order to restore thumb flexion or transfer of the extensor carpi radialis longus (ECRL) tendon to the FDP (index) tendon to restore index finger flexion.

## Discussion

Prehensile function, defined as the ability to hold, secure and manipulate an object with one hand, has been perfected by mankind. Arguably, thumb-to-index finger opposition may be one of the most important steps in human evolution. This is a two-step process that involves maximal pulp-to-pulp contact between the thumb and index finger and abduction/rotation of the thumb metacarpal at the trapezophalangeal joint (TMJ) of the thumb. The former involves index finger DIP joint flexion (FDP), index finger PIP joint flexion (FDS) and TMJ flexion by the opponens pollicis, a thenar muscle. Rotation of the TMJ involves the two other thenar muscles, the abductor pollicis brevis (APB), which cocks up the thumb for action, and the flexor pollicis brevis (FPB), which flexes and rotates the thumb. With a stronger force as in a "pinch grasp", the first dorsal interosseus (FDI), which can be easily palpated, is activated in assisting the FPB. The TMJ joint, a saddle joint, is hyper-mobile allowing abduction-adduction, flexion-extension, and medial-lateral rotation. This hyper-mobility allows thumb deftness with slender and fine muscles for stability, allowing precision and dexterity. This is a simplified view of thumb dynamics as the thumb is maneuvered by nine skeletal muscles. The median nerve is responsible for "precision grip" as in engaging a light bulb into its socket and is referred to as the "nerve of precision". This is in contradistinction to the ulnar nerve which is responsible for "power grip" as in kick-opening a jar and is labelled as "the nerve of power" [2].

One also needs to distinguish the "O" sign due to a median or AIN injury due to weakness of the FPL and FDP and inability to gesture the "okay sign" and the Froment sign due to ulnar nerve injury. In the latter, during a pinch maneuvre, a weak adductor pollicis is compensated for by activation of the FPL (hyperflexion of the thumb interphalangeal joint) and activation of the FDS to the index finger (hyperflexion of PIP joint) leading to an "okay gesture". This distinction is frequently confusing (Figure 3) [11]. 

Morton Spinner in his classic treatise on "Injuries to the Major Branches of Peripheral Nerves of the Forearm", outlines a comprehensive and graphic account of the various pathologies encountered with the AIN. Noteworthy is the lack of reported tumorous pathology. He also cautions the reader that AIN injury can be mimicked by rupture of tendons of the FPL and the FDP to the index finger as seen with rheumatoid arthritis and by congenital absence of these muscle groups in rare instances. Many of the etiologies include aberrant tendinous origins of muscles and accessory or variant muscles (Table 3) [1]. 

**Table 3:** Etiologies of the various diseases affecting the anterior interosseous nerve as outlined by Morton Spinner in his classic treatise [1].

ABERRANT TENDON ORIGIN	ACCESSORY MUSCLE/TENDON	VASCULAR	MUSCULO-SKELETAL	VARIANT MUSCLE	FRACTURE/TRAUMA	INFLAMMATORY
Deep head of pronator teres	Deep head of pronator teres	Thrombosis of crossing ulnar collateral vessels	Enlarged bicipital bursa near origin of anterior interosseus nerve	Palmaris profundus	Supracondylar humeral fracture	Parsonage-Turner syndrome
Flexor digitorum superficialis to the index finger	Accessory head of the flexor pollcis longus (Gantzer muscle)	Aberrant radial artery		Flexor carpi radialis brevis	Proximal radial fracture	Isolated neuritis of anterior interosseous nerve
		Enlarged median artery			Antebrachial venipuncture	Tendinitis of deep head of pronator trees
		Volkmann's ischemia			Stab or bullet wounds to proximal forearm	

The author has published a case of pseudo-AIN following herpes zoster infection complicated by a medial cord brachial plexopathy with the abnormal "O" sign and impaired precision grip [12]. Besides electromyographic denervation of the FPL, PQ, and FDP, the AIN motor nerve can be recorded from the FPL by nerve conduction study. The recording electrode can be placed over the belly of the FPL, with the reference electrode placed over the radial styloid, and the median nerve stimulated at the ante-cubital fossa. Normative values for CMAP amplitudes have been established for 50 controls [13].

The treatment of the AIN or pseudo-AIN syndrome depends on the etiology. An accurate diagnosis is critical. Acute onset of severe pain in the volar aspect of the upper forearm or shoulder girdle pain as an antecedent to weakness with the spread of weakness outside of the AIN territory is indicative of an isolated neuritis of the AIN or a Parsonage-Turner syndrome (brachial plexitis) respectively. Electrophysiological studies with nerve stimulation of the median nerve at the antecubital fossa with recording from the belly of the FPL, the triad of the variable weakness of the FPL, FDP, and PQ with acute denervation is a prerequisite. However, this is not a substitute for a thorough history and clinical examination, as outlined in the Introduction section, where we outlined the clinical pearls. When an inflammatory etiology is suspected, conservative measures are implemented with an empirical trial of a short course of prednisone (no clinical trials, empirical observation) and physical/occupational therapy [14]. Resolution may take up to 12 months or even longer. If the AIN lesion is pure, there is no antecedent severe pain or a constitutional prodrome and there is electrophysiological confirmation, then Morton Spinner advocates exploratory surgery after 12 weeks to look for an impingement or entrapment syndrome as outlined in Table 1. Refractory cases may be amenable to tendon transfer; a tendon slip from the FDS ring or middle finger tendon to the FPL tendon or FDP (index) tendon, on the assumption that the median nerve proper is intact. Other options include transfer of the brachioradialis (BR) tendon to the FPL tendon or the extensor carpi radialis longus (ECRL) tendon to the FDP (index) tendon [1].

Elbow debridements and synovectomies by arthroscopy are frequently performed in rheumatoid arthritis patients. This technique allows excellent visual access to the joints and facets of the elbow. However, the neurovascular bundles traversing the elbow joint can be relatively easy prey to the instruments of the surgeon and detailed knowledge of the anatomy of the region is essential [15,16]. Nevertheless, injuries to the nerves traversing the elbow are surprisingly rare. In a study of 222 peripheral nerve injuries following elbow arthroscopic surgeries, 32% were due to an ulnar nerve injury, 22% due to a radial nerve injury, 19% due to a posterior interosseus nerve injury. Only 23% of patients made a good recovery [17]. In a retrospective review of 473 elbow arthroscopies over an 18-year period, only 12 transient nerve palsies occurred: five ulnar nerve palsies, five superficial radial nerve palsies, one posterior interosseus nerve palsy, one medial antebrachial nerve palsy, and one AIN. These injuries were strongly associated with rheumatoid arthritis and elbow contracture [18]. In a retrospective survey of 560 elbow arthroscopies, there were eight ulnar, eight radial, one median, and three medial antebrachial cutaneous nerve palsies, all transient [19].

## Conclusions

The AIN and its origin, the median nerve, control dextrous finger and hand motion that epitomizes the evolutionary enhancement of a fundamental human trait, thumb-index opposition, deftness, and dexterity of the hand. Therefore these motions are complex and difficult to elucidate. Nevertheless, there are unique "spot diagnosis" patterns, such as executing the "O" gesture, which should betray the involvement of these nerves. Distinguishing an AIN and pseudo-AIN nerve injury should be relatively straightforward as the former involves exclusive weakness of a triad of muscles and the absence of sensory involvement. This seemingly straightforward clinical exercise is critical as the etiology of these entities may differ significantly, and as we outline in the Discussion section, the treatment approach can diverge accordingly.

## References

[1] Spinner M (1972). Injuries to the Major Branches of Peripheral Nerves of the Forearm. https://books.google.co.in/books/about/Injuries_to_the_Major_Branches_of_Periph.html?id=_F1sAAAAMAAJ&redir_esc=y.

[2] Napier J (1993). Hands. Hands.

[3] Bhadra N, Keith MW, Peckham PH (1999). Variations in innervation of the flexor digitorum profundus muscle. J Hand Surg Am.

[4] Chin DH, Meals RA (2001). Anterior interosseous nerve syndrome. J Hand Surg Am.

[5] Sood MK, Burke FD (1997). Anterior interosseous nerve palsy. A review of 16 cases. J Hand Surg Br.

[6] Ulrich D, Piatkowski A, Pallua N (2011). Anterior interosseous nerve syndrome: retrospective analysis of 14 patients. Arch Orthop Trauma Surg.

[7] Pham M, Bäumer P, Meinck HM, Schiefer J, Weiler M, Bendszus M, Kele H (2014). Anterior interosseous nerve syndrome: fascicular motor lesions of median nerve trunk. Neurology.

[8] Gunther SF, DiPasquale D, Martin R (1992). The internal anatomy of the median nerve in the region of the elbow. J Hand Surg Am.

[9] Spinner M (1970). The anterior interosseous-nerve syndrome, with special attention to its variations. J Bone Joint Surg Am.

[10] Sethi KS, Thompson LL (1989). The Electromyographer's Handbook. https://www.worldcat.org/title/electromyographers-handbook/oclc/19570261.

[11] Broussolle E, Rethy MP, Thobois S (2009). Jules Froment (1878-1946). J Neurol.

[12] Kesserwani H (2020). Post-herpetic brachial plexopathy: a rare case report with a side note on localizing brachial plexopathies and a literature review of post-herpetic segmental paresis. Cureus.

[13] Vucic S, Yiannikas C (2007). Anterior interosseous nerve conduction study: normative data. Muscle Nerve.

[14] Miller-Breslow A, Terrono A, Millender LH (1990). Nonoperative treatment of anterior interosseous nerve paralysis. J Hand Surg Am.

[15] Ruch DS, Poehling GG (1997). Anterior interosseus nerve injury following elbow arthroscopy. Arthroscopy.

[16] Voin V, Iwanaga J, Sardi JP, Fisahn C, Loukas M, Oskouian RJ, Tubbs RS (2017). Relationship of the median and radial nerves at the elbow: application to avoiding injury during venipuncture or other invasive procedures of the cubital fossa. Cureus.

[17] Desai MJ, Mithani SK, Lodha SJ, Richard MJ, Leversedge FJ, Ruch DS (2016). Major peripheral nerve injuries after elbow arthroscopy. Arthroscopy.

[18] Kelly EW, Morrey BF, O'Driscoll SW (2001). Complications of elbow arthroscopy. J Bone Joint Surg Am.

[19] Intravia J, Acevedo DC, Chung WJ, Mirzayan R (2020). Complications of elbow arthroscopy in a community-based practice. Arthroscopy.

